# Modeling tumor dynamics and predicting response to therapies in a murine pancreatic cancer model

**DOI:** 10.1038/s41540-025-00593-z

**Published:** 2025-11-04

**Authors:** Krithik Vishwanath, Hoon Choi, Mamta Gupta, Rong Zhou, Anna G. Sorace, Thomas E. Yankeelov, Ernesto A. B. F. Lima

**Affiliations:** 1 Oden Institute for Computational Engineering and Sciences, The University of Texas at Austin, Austin TX, USA; 2 Department of Aerospace Engineering and Engineering Mechanics, The University of Texas at Austin, Austin TX, USA; 3 Department of Mathematics, The University of Texas at Austin, Austin TX, USA; 4 Department of Radiology, The University of Pennsylvania, Philadelphia PA, USA; 5 Abramson Cancer Center, The University of Pennsylvania, Philadelphia PA, USA; 6 Department of Radiology, The University of Alabama, Birmingham, Birmingham AL, USA; 7 Department of Biomedical Engineering, The University of Alabama, Birmingham, Birmingham AL, USA; 8 Department of Biomedical Engineering, The University of Texas at Austin, Austin TX, USA; 9 Department of Diagnostic Medicine, The University of Texas at Austin, Austin TX, USA; 10 Livestrong Cancer Institutes, The University of Texas at Austin, Austin TX, USA; 11 Department of Imaging Physics, The University of Texas M.D. Anderson Cancer Center, Houston TX, USA; 12 Texas Advanced Computing Center, Austin TX, USA

**Keywords:** Biotechnology, Cancer, Oncology, Engineering, Mathematics and computing

## Abstract

We seek to establish a parsimonious mathematical framework for understanding the interaction and dynamics of the response of pancreatic cancer to the NGC triple chemotherapy regimen (mNab-paclitaxel, gemcitabine, and cisplatin), stromal-targeting drugs (calcipotriol and losartan), and an immune checkpoint inhibitor (anti-PD-L1). We developed a set of ordinary differential equations describing changes in tumor size under the influence of cocktails of treatments. Parameter estimation relies on three tumor volume measurements obtained over a 14-day period in a genetically engineered pancreatic cancer model (${{Kras}}^{{\rm{LSL}}-{\rm{G12D}}}\,;\,{{Trp53}}^{{\rm{LSL}}-{\rm{R172H}}}\,;\,{Pdx1}-{\rm{Cre}}$). Our model reproduces tumor growth in all scenarios with an average concordance correlation coefficient (CCC) of 0.99 ± 0.01. We conduct leave-one-out predictions (average CCC = 0.74 ± 0.06), mouse-specific predictions (average CCC = 0.75 ± 0.02), and hybrid, group-informed, mouse-specific predictions (average CCC = 0.85 ± 0.04). The developed mathematical model demonstrates high accuracy in fitting the experimental tumor data and a robust ability to predict tumor response to treatment. This approach has important implications for optimizing combination NGC treatment strategies.

## Introduction

Pancreatic cancer, infamous for its aggressive growth, early metastasis, and resistance to conventional therapies, necessitates innovative treatment approaches^1–3^. Emerging treatment protocols are increasingly focused on strategies that target not just the cancer cells, but also the tumor microenvironment and immune system^4–6^. Pancreatic tumors, known for their dense stromal tissue and immunosuppressive environments, present challenges for traditional chemotherapies like cisplatin and gemcitabine^7–12^. To address this, stromal-targeting agents such as calcipotriol and losartan are being investigated to enhance drug delivery, while immunotherapies, such as anti-PD-L1 (anti-Programmed Cell Death Ligand 1), are being tested to activate the immune system against the tumor^5,13–15^. However, determining the optimal combination of these therapies and predicting tumor response remains an active area of research.

The literature on mathematical modeling in pancreatic cancer is sparse, especially when compared to other cancers^16,17^. However, there are seminal efforts to model the efficacy of chemotherapy^18,19^ and immunotherapy^20,21^ in pancreatic cancer. In ref. ^18^, Lee et al. examined the inconsistent responses of pancreatic cancer to gemcitabine observed between the in vitro (high efficacy) and in vivo settings (low efficacy). They used a system of partial differential equations to model cell proliferation, apoptosis, and nutrient diffusion gradients influenced by the microenvironment (e.g., inefficient vascularization or abundant stroma). The model, using parameters estimated with in vitro data on drug exposure and cell viability and validated through in vivo mouse experiments, indicates that pancreatic tumors essentially resist gemcitabine due to poor vascularization. The study shows that the drug’s efficacy in controlled in vitro conditions, which mimic a single cell layer near the vasculature with optimal access to oxygen and nutrients, does not capture the complex dynamics of in vivo environments. In another work, Jenner et al.^19^ used a hybrid agent-based model to evaluate the ability of locally delivered gemcitabine to treat pancreatic cancer effectively. The model was estimated using both in vitro (drug release kinetics and cytotoxicity against human pancreatic cancer cells from gemcitabine-loaded alginate fibers) and in vivo (including tumor growth rates and responses from mouse experiments) data. In their approach, the authors specifically investigated how the tumor microenvironment, including factors like drug diffusion and cell proliferation rates, impacts the effectiveness of drug delivery. Their findings demonstrated that intratumoral placement of drug-loaded alginate fibers, which accounts for these microenvironmental factors, significantly improved treatment efficacy compared to peritumoral placement. They found that the drug release rate and pattern—such as constant, exponential, and sigmoidal releases—significantly influenced the drug’s ability to maintain therapeutic concentrations within the tumor microenvironment over time, with the exponential profile proving more effective in reducing tumor growth than others. This indicates that fine-tuning the release profile could be critical for optimizing treatment responses in pancreatic cancer.

Building on the investigation of drug delivery strategies within the complex pancreatic tumor microenvironment, other researchers have focused on understanding the interactions between pancreatic cancer cells and the immune system, which are crucial for improving patient survival. A system of five ordinary differential equations (ODEs) was developed by Hu et al. to investigate the dynamics of pancreatic cancer cells, their interactions with the immune system, and how this impacts patient survival^21^. This system models the interactions between pancreatic cancer cells, stellate cells, effector cells, and both tumor-promoting and tumor-suppressing cytokines. The model, which integrates 23 parameters sourced from the literature, was validated using survival data from two clinical trials. Findings based on optimal control theory indicated that mono-immunotherapy alone cannot effectively control pancreatic cancer, suggesting the necessity for combined therapies, including anti-TGF-*β* treatments and adoptive transfers of immune cells (a process where immune cells are harvested, sometimes genetically modified, and then infused back into the patient to boost the immune response), to enhance patient survival. Bratus et al. also employed an ODE framework to simulate the dynamics of cancer cell mutations and their interactions with CD8 T cells and nutrients^20^. The model describes the temporal dynamics of various pancreatic cancer cell populations differentiated by specific genetic mutations and includes mutation and fitness landscape matrices that characterize the cells’ survival capabilities. The model was able to effectively predict the growth dynamics of pancreatic cells with different mutations and their response to immune cells, notably demonstrating the effectiveness of immune cells in reducing tumor size. The authors did note, however, that incorporating experimental data into their study would improve the model’s validity.

In this contribution, our goal is to develop a hierarchical framework capable of simulating and predicting the response of pancreatic tumors to various combination treatment regimens. While previous studies have provided valuable insights into the interactions between pancreatic cancer cells, the immune system, and drug delivery within the tumor microenvironment, they often focus on specific aspects, such as modeling the mutation dynamics within pancreatic cancer cells. Building on these foundational efforts, our work seeks to provide a comprehensive model that integrates the dynamics of chemotherapy, stromal-targeting drugs, and immunotherapy. To our knowledge, this represents the first effort to mathematically model the dynamics of NGC chemotherapy effects on pancreatic cancer. Specifically, we aim to model the dynamics of tumor growth and regression in response to combinations of chemotherapy, stromal-targeting drugs, and immunotherapy. By integrating experimental data from longitudinal tumor volume measurements obtained in murine models of pancreatic cancer, we develop a mathematical model that reproduces bulk tumor growth and regression under multiple treatment regimens. We further demonstrate the ability of this model to predict mouse-specific responses to treatments. This study contributes to a biology-based, mathematical model that simulates complex treatment interactions and predicts responders and non-responders, which is crucial for optimizing personalized treatment strategies in pancreatic cancer.

**Fig. 1:**
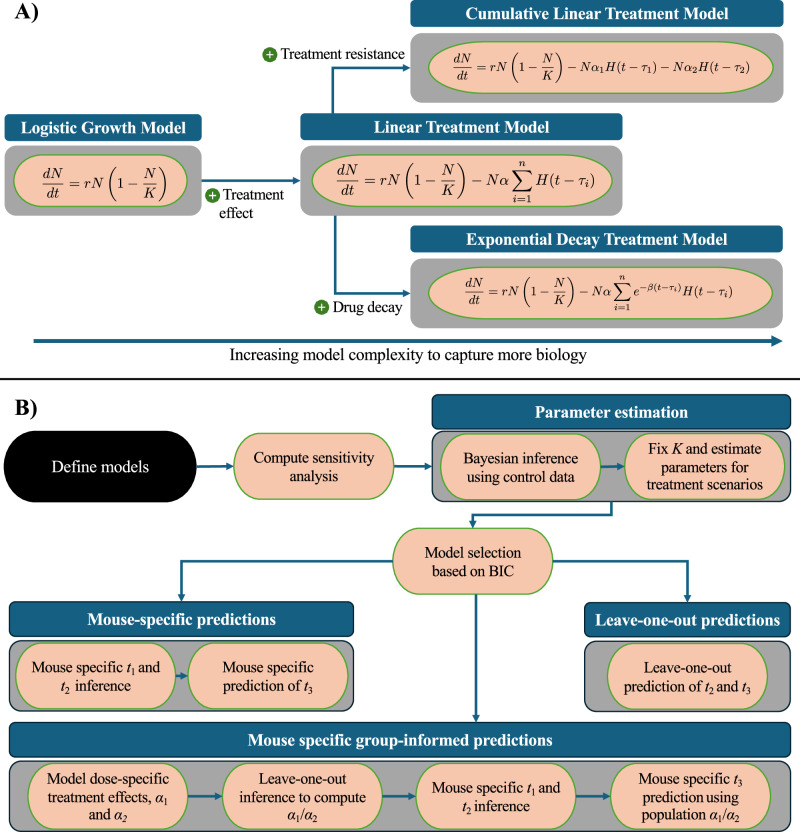
Illustration of the model building, parameter estimation, and prediction framework. **A** Our models are built from a logistic growth function. To develop our Linear Treatment Model, we add a linear treatment effect for each treatment week. For our Cumulative Linear and Exponential Decay treatment models, we also include in the effects of treatment resistance (i.e., variable treatment intensity across administrations) and drug decay, respectively. **B** The process begins with the definition of mathematical models designed to parsimoniously describe the treatment scenarios. Sensitivity analyses are then performed to determine the relative impact of each model parameter. After estimating parameters of the control and treatment data to each model, the Bayesian Information Criterion (BIC) guides the selection of a single “best” model for predictions. *t*_*i*_ and *α*_*i*_ refer to the *ith* time of tumor volume measurement and *ith* tumor death rate due to treatment, respectively. Using the chosen model, three prediction scenarios are investigated: 1) leave-one-out predictions for days 7 and 14 tumor volumes, 2) mouse-specific parameter estimations using the days 0 and 7 data to predict the day 14 tumor volumes, and 3) mouse-specific, predictions made by incorporating the population’s average resistance to the treatment ($\frac{{\alpha }_{1}}{{\alpha }_{2}}$) when estimating parameters using the day 0 to day 7 data to predict day 14 tumor volumes.

To unite these modeling efforts into a single, coherent workflow, we propose a hierarchical, treatment-agnostic framework that (i) defines simple ODEs for tumor growth and treatment effects, (ii) performs global sensitivity analyses to prioritize key parameters, (iii) uses Bayesian parameter estimation to fit control and treatment data, and (iv) generates individualized and population-informed predictions of treatment response (Fig. 1).

## Results

### Estimation of logistic model parameters using the control group

Following the workflow outlined in Fig. 1, we first employ the priors defined in Table 1 to estimate the proliferation rate (*r*), carrying capacity (*K*), and initial condition (*N*_0_) from the control data. These priors were selected based on prior predictive checks, which confirmed (via a coverage metric of 100%, see Supplemental Figures) that the sampled parameter combinations generate tumor growth dynamics consistent with the observed control data, thus supporting their biological plausibility and suitability for inference.

**Table 1:** Parameter definitions and uniform priors for inference of treatment arm parameters

Parameter	Meaning	Prior
*N*_0_	initial tumor volume	${\mathcal{U}}(0,600)$ mm^3^
*r*	tumor proliferation rate	${\mathcal{U}}(0.03,0.3)$ d^−1^
*α*	tumor death rate by treatment	${\mathcal{U}}(0,0.3)$ d^−1^
*β*	treatment effect decay	${\mathcal{U}}(0,2)$

${\mathcal{U}}(a,b)$ denotes a uniform distribution with bounds *a* and *b* for the prior values of the parameter. These bounds are determined using prior predictive checks with the control data. For the control, we assume that the proliferation rate *r* is uniformly distributed over (0, 0.5) d^−1^, the carrying capacity *K* over (1, 3000) mm^3^, and the initial volume *N*_0_ over (0, 600) mm^3^. We also note that the bounds for the proliferation rate for the treatment arms are based on the posterior distribution of the control for that parameter.

We model tumor volume dynamics using the general treatment-agnostic formulation:

1 $$\frac{dN}{dt}=rN\left(1-\frac{N}{K}\right)-N\mathop{\sum }\limits_{i = 1}^{n}{\alpha }_{i}{e}^{-\beta (t-{\tau }_{i})}H(t-{\tau }_{i}),$$

where *N*(*t*) is the tumor volume at time *t*, *α*_*i*_ represents the death rate due to the *ith* treatment dose, *τ*_*i*_ is the time of administration, *β* is the decay rate of treatment effect, and *H*(*t* − *τ*_*i*_) is the Heaviside step function. For the control group, we assume *α*_*i*_ = 0 and *β* = 0, which simplifies Eq. (1) to the standard logistic growth model.

**Fig. 2:**
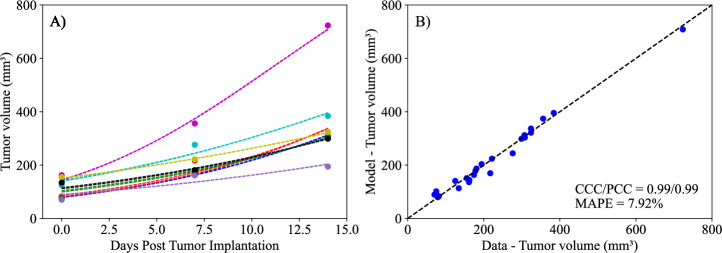
Parameter estimation for control data to the logistic model described by Logistic Growth Model, Eq. (2). **A** Presents the fitting of the model to individual mouse tumor volumes (solid points) at three time points (days 0, 7, and 14) over the two-week experimental period, along with their corresponding estimated curve (dashed lines). **B** Compares the experimentally measured tumor volume and the model’s median posterior value. The dashed black line in Panel B is the line of unity. The fitted logistic model demonstrates exceptional alignment with the control data, achieving a concordance correlation coefficient (CCC) and a Pearson correlation coefficient (PCC) of 0.99 each, along with a mean absolute percent error (MAPE) of 7.92%. As the control group received no treatment, by day 14 all mice exhibit a significantly higher final tumor volume compared to their initial tumor volume on day 0 (two-tailed paired t-test, *p* = 0.0013).

In this scenario, we estimate a population-specific *K*, along with a mouse-specific *r* and *N*_0_ (i.e., each mouse having its own distribution of *r* and *N*_0_). As depicted in Fig. 2, the logistic model (i.e., Logistic Growth Model, Eq. (1) with *α*_*i*_ = 0 and *β* = 0) can accurately describe the experimental data from the control group, with a concordance correlation coefficient (CCC) and Pearson correlation coefficient (PCC) of 0.99 when comparing the experimental and estimated tumor volumes, with a mean absolute percent error (MAPE) below 8%.

Moving forward, we use these parameters when estimating parameters of the Linear Treatment Model and the Exponential Decay Treatment Model, which are special cases of Eq. (1). The Linear Treatment Model corresponds to setting *β* = 0 and assuming *α*_1_ = *α*_2_ = *α*, while the Exponential Decay Treatment Model involves estimating *β* (i.e., allowing decaying treatment effects), again with *α*_1_ = *α*_2_ = *α*. In both models, *n* = 2 since treatment is administered at two time points. A summary of the assumptions for each model is provided in Table 2. Specifically, the prior distribution for the Bayesian parameter estimation of *r* for each mouse in the treatment groups is established using the upper and lower bounds determined from the posterior distribution of the control group’s proliferation rate. Additionally, the carrying capacity calculated from the control group, *K*, is set to the median value from this posterior distribution when estimating the treated groups’ parameters. This allows us to reduce the number of parameters requiring estimation in the other models and mitigate potential issues with parameter identifiability.

**Table 2:** Summary of tumor growth models used in this study

Model (Eq.)	*β*	*α*_1_	*α*_2_	Notes
**Logistic Growth Model** (Eq. (2))	0	0	0	Control data only
**Linear Treatment Model** (Eq. (3))	0	*α*	*α*	Constant treatment effect
**Exponential Decay Treatment Model** (Eq. (4))	*β*	*α*	*α*	Decaying treatment effect
**Cumulative Linear Treatment Model** (Eq. (10))	0	*α*_1_	*α*_2_	Dose-specific treatment effect

Each model corresponds to a special case of Eq. (1) with specific parameter settings, as listed below.

### Parameter estimation of treatment-based models

Following the model fitting of the Logistic Growth Model (i.e., Eq. (1) with *α*_*i*_ = 0 and *β* = 0) to the control data, we proceed to estimate the parameters in the Linear Treatment Model (i.e., Eq. (1) with *β* = 0 and *α*_1_ = *α*_2_ = *α*) and the Exponential Decay Treatment Model (i.e., Eq. (1) with estimated *β* and *α*_1_ = *α*_2_ = *α*) using the data from each treatment protocol. In both models, we estimate the parameters *r*, *α*, and *N*_0_ for each mouse, while fixing the carrying capacity to the value obtained from fitting the control data to the Logistic Growth Model. This pragmatic decision to fix *K*, while necessary given the limited data during the exponential phase, also means that models which might otherwise benefit from estimating *K* are potentially penalized under the Bayesian Information Criterion (BIC) due to reduced flexibility in capturing the full logistic curve. This trade-off between parameter identifiability and model flexibility should be considered when interpreting the BIC differences between the models. Additionally, the Exponential Decay Treatment Model requires the parameter estimation of *β* (i.e., the treatment decay effect) as a population-based parameter, with each treatment protocol having its own distribution of *β*. Notably, the models in consideration are treatment agnostic and do not depend on the specific nature of any individual treatment combination.

**Fig. 3:**
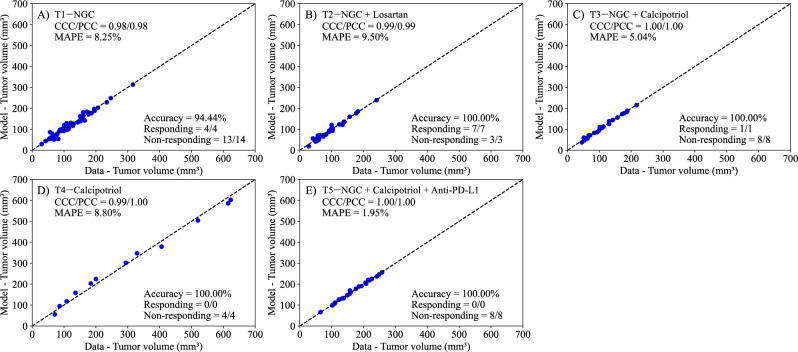
Comparison between the experimentally measured tumor volume and inference of logistic growth with a linear treatment term (Linear Treatment Model, Eq. (3)) for each treatment protocol. Panels **A**–**E** correspond to the following treatment protocols: **A**) NGC, **B**) NGC+ Losartan, **C**) NGC + Calcipotriol, **D**) Calcipotriol, and **E**) NGC + Calcipotriol + Anti-PD-L1 mAb. The dashed black lines are the line of unity. All parameter estimations to treatment scenarios exhibit high levels of correlation between the data and model, with all CCCs and PCCs greater than 0.98. The average accuracy, calculated as the number of correctly identified responders and non-responders divided by the total number of mice for each treatment, was 98.89 ± 1.11% across all treatment scenarios. Further, the MAPE for allscenarios is less than 10%. This suggests that the Bayesian estimation of model parameters (*r*, *α*, and *N*_*0*_) effectively reproduces the experimental data.

**Fig. 4:**
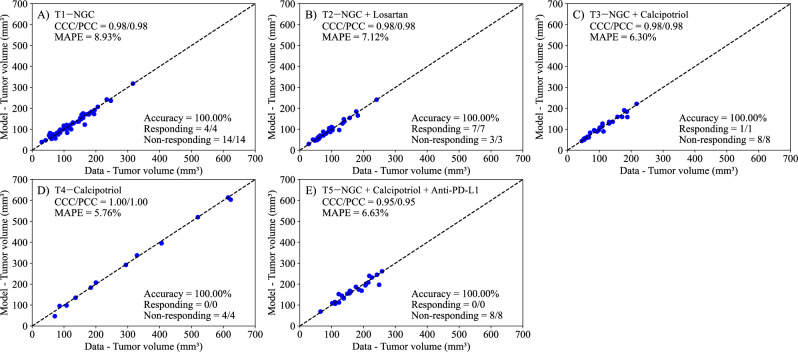
Comparison between the experimentally measured tumor volume and posterior predictive inference of logistic growth with a drug decay treatment term (Exponential Decay Treatment Model, Eq. (4)) for each treatment protocol. Panels **A**–**E** correspond to the following treatment protocols: **A**) NGC, **B**) NGC + Losartan, **C**) NGC + Calcipotriol, **D**) Calcipotriol, and **E**) NGC + Calcipotriol + Anti-PD-L1 mAb. The dashed black lines are the line of unity. All parameter estimations to treatment scenarios exhibit high levels of correlation between the data and model, with all CCCs and PCCs greater than 0.95. The average accuracy was 100% across all treatment scenarios. Further, the MAPE for all scenarios is less than 10%. This suggests that the Bayesian parameter estimation of model parameters (*r*, *α*, *β*, and *N*_*0*_) effectively reproduces the experimental data.

In Fig. 3, we present the experimental data and the model solutions for all five treatment scenarios. The model differentiates between responders and non-responders with 100% accuracy in all treatments except T1—NGC, where the model achieved a 94.44% accuracy. The MAPE for the tumor volume is below 10%, and the CCC/PCC is above 0.98 for all treatment protocols. Similarly, we display the results for the Exponential Decay Treatment Model in Fig. 4. This model can differentiate between responders and non-responders with an accuracy of 100% in all scenarios except treatment protocols 1 and 5, where the model achieved an accuracy of 94.44% and 87.50%, respectively. Notably, the only discernible difference in terms of accuracy between the two treatment-agnostic models occurs in T5—NGC + calcipotriol + anti-PD-L1, where the Exponential Decay Treatment Model incorrectly classifies one non-responder as a responder. The CCC/PCC for the second model is above 0.95 for all treatment protocols, and the MAPE for tumor volume is below 10% for all treatment scenarios.

### Model selection

In Table 3, we present the BIC for each model (linear and exponentially decaying) for each treatment protocol. Note that a lower BIC indicates a better fit of the model to the data^22^. Our results show that the Linear Treatment Model (i.e., Eq. (1) with *β* = 0 and *α*_1_ = *α*_2_ = *α*) has a lower BIC in all treatment protocols than the Exponential Decay Treatment Model (with estimated *β*). Therefore, this model is selected for the subsequent analyses in this study.

**Table 3:** Calculated Bayesian Information Criterion (BIC) on parameter estimation for parameters in the Linear Treatment Model and the Exponential Decay Treatment Model

Treatment protocol	Treatment term assumption	
	Linear, Eq. (3)	Exponential decay, Eq. (4)
T1—(NGC)	1198	1244
T2—(NGC+losartan)	640	662
T3—(NGC+calcipotriol)	540	601
T4—(calcipotriol)	246	261
T5—(NGC+calcipotriol+anti-PD-L1)	469	555

See Fig. 1 for the list of the drugs included within each treatment protocol.

### Parameter distributions of parameter estimation

**Fig. 5:**
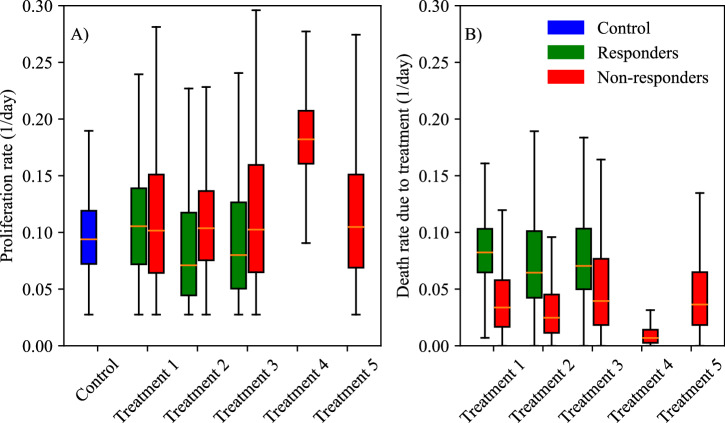
Box and whisker plots of posterior parameter distributions for each treatment protocol. Specifically, parameter distributions for proliferation rate (*r*) and death rate due to treatment (*α*) are displayed in (**A**) and (**B**), respectively. Bayesian distributions are displayed for control (blue) and each treatment, split into responders (green) and non-responders (red). Responders exhibit statistically lower proliferation rates and greater death rates due to treatments (*p*-adjusted < 0.001). In particular, T2—NGC + losartan and T3—NGC + calcipotriol induce the greatest death rate due to the regimes. T4—calcipotriol performed the worst, with both the highest tumor proliferation rate and lowest death rate due to treatment. Note that neither T4—calcipotriol nor T5—NGC + calcipotriol + anti-PD-L1 had any responders.

After selecting Linear Treatment Model as the best model to represent the data (according to the BIC), we proceed to examine the posterior parameter distributions for each of the control and treatment protocols. Figure 5 presents significant differences across all scenarios for the tumor proliferation rates, *r*, and tumor death rate due to treatment, *α*. In Panel A), the median proliferation rate for non-responders is significantly higher than that for responders across T1—NGC, T2—NGC + losartan, and T3—NGC+calcipotriol (23.44 ± 14.50% higher than responders, *p*-adjusted < 0.001). T4—calcipotriol has the highest proliferation rate, while T2—NGC + losartan and T3—NGC+calcipotriol have the lowest. Except for the responders in T2—NGC + losartan and T3—NGC+calcipotriol, all treatment groups have a median proliferation rate that significantly surpasses the control group. Panel B) displays the distribution of the parameter for the death rate due to treatment. In scenarios with both responders and non-responders (i.e., T1—NGC, T2—NGC + losartan, and T3—NGC+calcipotriol), the median death rate of non-responders is 54.8 ± 5.54% lower (*p*-adjusted < 0.001) than that of responders. Statistical analysis using a Bonferroni-adjusted Mann-Whitney U test indicates that differences between all pair-wise groups (i.e., responders and non-responders) in terms of tumor proliferation rates and tumor death rates due to treatment are significant (*p*-adjusted < 0.001). The lowest death rate due to treatment is associated with T4—calcipotriol, while the highest is observed in the responders in T1—NGC.

### Parameter estimation of model with cumulative drug effect

**Fig. 6:**
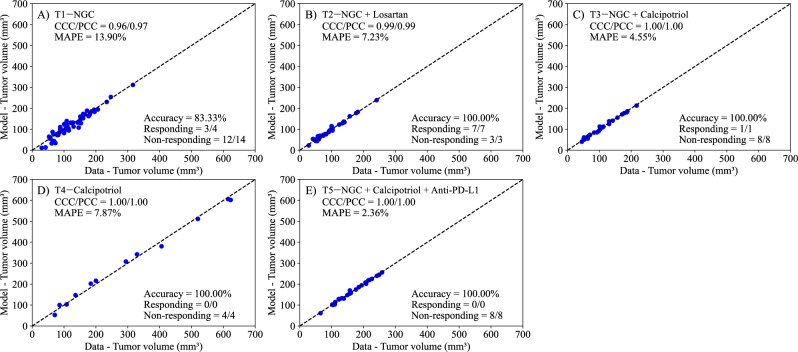
Comparison between experimentally measured tumor volume and parameter estimation of logistic growth with two linear treatment terms (Eq. (3)). Panels **A**–**E** correspond to the following treatment protocols: **A**) NGC, **B**) NGC + Losartan, **C**) NGC + Calcipotriol, **D**) Calcipotriol, and **E**) NGC + Calcipotriol + Anti-PD-L1 mAb. The model now includes the effects from two separate treatments death rates (*α*_1_ and *α*_2_) to account for the individual effects of both treatment days. The dashed black lines are the line of unity. All treatment scenarios exhibit high levels of correlation between the data and the model, with all CCCs and PCCs greater than 0.96. The average accuracy was 96.67 ± 3.33% across all treatment scenarios. Further, the MAPE for all scenarios is less than 14%. This suggests that the Bayesian parameter estimation of model parameters (*r*, *α*_1_, *α*_2_, and *N*_0_) effectively reproduces the experimental data.

In this parameter estimation, our goal is to quantify the compounding effect of the treatment regime by estimating a different *α* at each of the two intervals of treatment administration, effectively separating the death rate due to treatment into *α*_1_ and *α*_2_ (corresponding to the death rates caused by the first and second administration intervals of treatment, respectively). We estimate parameters *r*, *α*_1_, *α*_2_, and *N*_0_ from the experimental data, and the results are presented in Fig. 6. The Cumulative (Two-Dose) Linear Treatment Model is a modified version of the Linear Treatment Model, corresponding to Eq. (1) with *β* = 0 and *α*_1_ ≠ *α*_2_. This model successfully captures the experimental data, with a CCC/PCC greater than 0.96 for all treatment scenarios. Further, the model differentiates between responders and non-responders with a 100% accuracy in all scenarios except T1—NGC, where the accuracy is 83.33%.

**Fig. 7:**
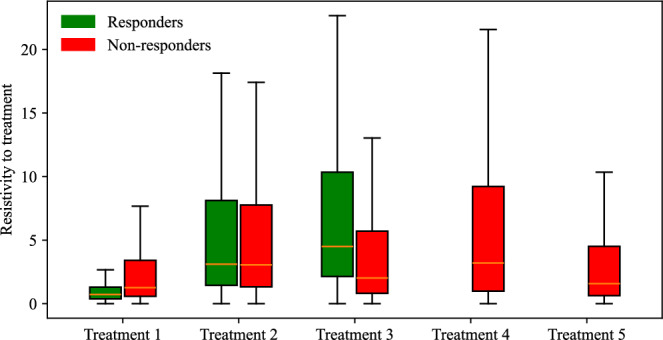
Box and whisker plots indicating the range of resistivity values encountered across distinct treatment scenarios. Each treatment scenario’s posterior distribution for resistivity to treatment, split into responders (green) and non-responders (red), is presented. The magnitude of treatment resistivity represents the ratio of the effect of the first dose (*α*_1_) to the effect of the second dose (*α*_2_); i.e., ($\frac{{\alpha }_{1}}{{\alpha }_{2}}$). For all groups, the interquartile range is between 1 and 10, indicating a significantly lower efficacy of the second dose compared to the first. Statistical analysis using the Bonferroni-adjusted Mann-Whitney U test shows that the differences in treatment resistivity between groups are significant (*p*-adjusted < 0.001). Out of all scenarios, T1—NGC has the lowest resistivity to treatment.

Due to the presence of only two treatment administration intervals in each protocol, we assume the compounding effect occurs in a linear fashion; that is, we model each treatment as directly proportional to the previous treatment administration interval by a constant, defined as treatment resistivity ($\frac{{\alpha }_{1}}{{\alpha }_{2}}$). According to this definition, a higher treatment resistivity indicates a decrease in impact for the second dose of treatment relative to the first (e.g., a ratio of 2 means the first dose is twice as effective as the second). Our findings reveal that across all treatment protocols, the median resistivity to treatment is between 0.72 and 4.50, indicating that the first delivery of treatment is between 0.72 and 4.5 times more effective than the second delivery of treatment. Figure 7 presents the posterior distribution of ($\frac{{\alpha }_{1}}{{\alpha }_{2}}$) for each group. Of all treatment protocols, the responders and non-responders in treatment protocol 1 have significantly lower resistance to treatment (median 0.72 and 1.26, respectively; *p*-adjusted < 0.001), while responders in T3—NGC + calcipotriol and non-responders in T4—calcipotriol exhibit the highest resistance to treatment (4.50 and 3.2, respectively; *p*-adjusted < 0.001).

### Model predictions

Following the framework presented in Fig. 1, the next step is to predict tumor volumes using our selected model. We apply three prediction scenarios: leave-one-out predictions, mouse-specific predictions, and group-informed, mouse-specific predictions (see Supplemental Figs. 1–3 for detailed visualization of model predictions). The first prediction scheme involves mouse-specific predictions, where the parameters *r*, *α*, and *N*_0_ are estimated to each mouse using the data from days 0 and 7. Following this parameter estimation, we predict the tumor volume at day 14. In Table 4’s Mouse Specific Row, we compare the model predictions to the true experimental data on day 14. The model successfully differentiates responders and non-responders 70.50 ± 10.53% across all treatments, with both the CCC and PCC surpassing 0.68 for every treatment condition. The MAPE remained under 18.55% for each scenario, showing the best precision for T5—NGC + calcipotriol + anti-PD-L1 with a MAPE of 11.96%, while T2—NGC + losartan had the highest error at 18.55%. Overall, the model underpredicts the final mice volume and had higher rates of successfully predicting responders (12/12) rather than non-responders (20/37). This observation may be attributed to the model’s parameters being estimated only for the first 7 days, potentially indicating differences in treatment efficacy during the second period of the treatment regimen (from day 7 to day 14). Inspection of the raw trajectories (Supplemental Fig. 4) shows that 39% (18/47) of tumors exhibit a reversal in the direction of growth or shrinkage from day 0 to 7 and from day 7 to 14.

**Table 4:** Evaluation of performance metrics for prediction methods

Approach / Metric		T1	T2	T3	T4	T5	Average
**Mouse-Specific**	Accuracy (%)	55.56	90.00	44.44	100.00	62.50	70.50
	CCC	0.72	0.77	0.79	0.79	0.68	0.75
	PCC	0.74	0.82	0.83	0.93	0.72	0.81
	MAPE (%)	14.51	18.55	13.84	11.78	11.96	14.13
**Leave-One-Out**	Accuracy (%)	77.78	10.00	88.89	100.00	87.50	72.83
	CCC	0.70	0.54	0.79	0.92	0.73	0.74
	PCC	0.73	0.56	0.87	0.92	0.73	0.76
	MAPE (%)	27.75	32.72	19.41	17.24	14.46	22.32
**Group-Informed**	Accuracy (%)	55.56	90.00	77.78	100.00	87.50	82.17
	CCC	0.72	0.92	0.90	0.92	0.77	0.85
	PCC	0.75	0.92	0.91	0.97	0.78	0.87
	MAPE (%)	14.23	11.88	9.61	7.39	9.50	10.52

Full prediction results by scenario are depicted visually in Supplemental Figs. 1–3.

The second prediction scheme, leave-one-out predictions, work by utilizing only the first time point and the group-informed parameter samples. Through this scheme, the model can differentiate between responders and non-responders with an average accuracy 72.83 ± 16.10% across all treatment protocols. Additionally, for all cases except for T2—NGC + losartan (NGC Backbone with Losartan), both the CCC and PCC are above 0.7. The MAPE remains below 33% across all scenarios, with the smallest percent error observed in T5—NGC + calcipotriol + anti-PD-L1 (MAPE = 14.75%) and the highest in T2—NGC + losartan (MAPE = 32.22%).

Our final prediction scenario, the group-based, mouse-specific prediction method, builds upon the mouse-specific prediction by incorporating into the model the diminishing effectiveness of repeated treatments, as illustrated in Fig. 7. Similar to the first prediction scheme, we calculate group-averaged resistance to treatment ($\frac{{\alpha }_{1}}{{\alpha }_{2}}$) within each treatment protocol. We use this value to adjust the posterior distribution of the individual mouse’s *α*_1_ and estimate *α*_2_. This is then integrated as the new predicted death rate of our second treatment dose to predict treatment effects during the second day of treatment. Similar to our mouse-specific prediction scheme, the parameters are estimated using the tumor volume data from days 0 to 7, and the model then predicts the final tumor volume on day 14. The outcomes of our group-based, mouse-specific predictions are presented in the final row of Table 4. This integrated method successfully distinguishes between treatment responders and non-responders, achieving an average accuracy of 82.17 ± 7.53% across all treatment scenarios. Moreover, the prediction model attains a CCC/PCC of over 0.9 for T2—NGC+losartan, T3—NGC+calcipotriol, and T4—calcipotriol, accurately reflecting the actual experimental outcomes for these groups. For T1—NGC and T5—NGC+calcipotriol+anti-PD-L1, the model achieves a CCC/PCC of over 0.72. Lastly, the MAPE remains below 15% for all scenarios, with the best performance observed in T4—calcipotriol (7.29%) and the least favorable in T1—NGC (14.32%).

## Discussion

We developed two mathematical models, one with linear treatment effects (i.e., assuming *β* = 0) and one with exponentially decaying treatment effects (with *β* estimated), to characterize and predict the response of pancreatic tumors to six combinations of chemotherapy, stromal-targeting drugs, and immunotherapy. As both models are identical in the absence of treatment (representing logistic tumor growth), we first estimated parameters of the control group, achieving a CCC of 0.99 (Fig. 2), and used the carrying capacity value obtained here when estimating parameters of the two models. Although a fully Bayesian approach using an informative prior for the carrying capacity (*K*) would be more consistent with a comprehensive uncertainty quantification framework, we opted to fix *K* at the value estimated from the control group. This decision was motivated by the limited number of datapoints per mouse, which made reliable parameter identifiability challenging when *K* was treated as a free parameter in each treatment-specific scenario. We recognize that, in principle, a tightly concentrated prior—derived from the posterior distribution of the control group—could be implemented in the treatment group analyses to propagate uncertainty in *K*. In future work, and with richer datasets, adopting such a Bayesian strategy might offer additional insights into the robustness of the model predictions. The parameter estimation of the linear model provided a CCC of 0.99 ± 0.01 across the five treatment groups (Fig. 3), while the exponentially decaying model resulted in a CCC of 0.98 ± 0.02 (Fig. 4). After parameter estimation, the BIC selected the linear treatment model as the most parsimonious (Table 3). This model demonstrated a high degree of accuracy in fitting the in vivo data, effectively capturing the complex interactions between tumor cells, stromal components, and immune cells, allowing for robust predictions of tumor growth and regression.

To leverage published biological knowledge while remaining transparent to regulators and clinicians, we estimate all model parameters via Bayesian MCMC using informative priors—thereby avoiding biologically implausible fits and fully propagating uncertainty—and then assess model-fit and hypothesis tests with standard frequentist diagnostics (e.g., p-values, BIC). This “best-of-both-worlds” strategy is well established in oncology and aligns with current FDA/EMA guidance on preclinical-to-clinical translation, ensuring both rigorous uncertainty quantification and familiar error-control for our translational audience^23–28^. Moreover, our ICC and CV analyses confirm that initial burden, proliferation rate, and treatment-effect parameters exhibit high inter-subject variability, whereas the noise term does not—further justifying individualized parameter estimation to capture true tumor heterogeneity (Supplemental Table 1).

According to the results from the sensitivity analysis (Supplemental Fig. 5), in the model with exponentially decaying treatment effects, both the death rate due to treatment, *α*, and the decay rate of the treatment effect, *β*, have a negligible effect on the tumor volume compared to other parameters, such as the carrying capacity and proliferation rate. The parameter ranges used in the sensitivity analysis were selected to capture the full range of possible outcomes, from scenarios where the treatment has no effect on the tumor to those where the tumor is eliminated. Even within these wide parameter ranges, *α* and *β* remained less influential on the final tumor volume. However, in the linear treatment model, where the effect of treatment does not diminish over time, *α* becomes the most influential parameter in determining the final tumor volume. This outcome is expected, given that the range of *α* used in both models is the same, and the inclusion of decay in the exponential model reduces the impact of *α* on tumor volume. However, we note that with the addition of an *α*-adjusted prior for the exponential model, the impact of the *α* term becomes significantly more influential in this model.

Our approach builds on the familiar logistic growth assumption of common tumor growth inhibition models (such as Claret et al.^29^) but introduces several key innovations. Unlike traditional tumor growth inhibition models, our formulation is treatment-agnostic, capturing overall tumor response without explicit PK/PD modeling of individual agents. Additionally, it explicitly accounts for multiple treatment doses—allowing for constant or decaying effects and differentiating successive doses with separate parameters—while also incorporating a treatment resistivity metric ($\frac{{\alpha }_{1}}{{\alpha }_{2}}$) to assess cumulative treatment efficacy. These enhancements offer a more flexible framework for simulating tumor trajectories and understanding combination therapy dynamics in preclinical settings.

We utilized Monte Carlo Markov Chains to estimate parameter posterior distributions for each individual mouse across all groups in a mixed-effects approach. Treatment protocols of T1—NGC, T4—calcipotriol, and T5—NGC + calcipotriol + anti-PD-L1 all expressed a higher proliferation rate than the control. In our model, the effective proliferation rate (*r*) not only captures intrinsic cell division, but also the net effect of various biological processes, including those influenced by treatment. The higher proliferation rates observed in T1—NGC (chemo cocktail), T4—calcipotriol (stromal-targeting drug), and T5—NGC+calcipotriol+anti-PD-L1 (chemo+stromal+immune) are not necessarily contradictory to the intended cytostatic or cytotoxic effects of these treatments. For example, it is apparent from the tumor progression data itself that mice in T4—calcipotriol experience more aggressive tumor growth than those in the control group, with the tumor volume increasing on average by 458% over 14 days compared to a 232% increase in the control group (Supplemental Fig. 4). This difference naturally leads to a higher proliferation rate in the model. Although no chemotherapy is administered in this protocol, the stromal-targeting drug can disrupt the tumor microenvironment. This disruption may eliminate subpopulations of cancer-associated fibroblasts that (under certain conditions) actually act to restrain tumor growth. That is, by removing these inhibitory stromal components, the drug may actually result in a more proliferative tumor phenotype^30,31^.

Next, we employed the linear model to predict tumor volume at the third time point using three different prediction scenarios: leave-one-out prediction, mouse-specific prediction, and a hybrid group-informed, mouse-specific prediction. Of these methods, the best-performing method was the hybrid method, with an average CCC of 0.85 ± 0.09. This method was able to successfully differentiate between treatment responders and non-responders by propagating the model forward with an average accuracy of 81.26 ± 19.03% across all treatment scenarios. In more detail, the leave-one-out method correctly predicted only 1 out of 12 responders and 33 out of 37 non-responders, while the mouse-specific method performed better in predicting responders (12/12) but only correctly identified 20 out of 37 non-responders. The hybrid group-informed, mouse-specific prediction method was able to identify 11 out of 12 responders and 26 out of 37 non-responders, highlighting that the effect of the treatment indeed differs with subsequent administrations. In the hybrid method, by splitting up the effects of the treatment based on individual time points (i.e., splitting *α* into *α*_1_ and *α*_2_), we isolate and evaluate the effect of combining treatments. To obtain the median resistivity (i.e., $\frac{{\alpha }_{1}}{{\alpha }_{2}}$) we first estimate parameters of the model using the entire dataset (Fig. 6), and then apply this median value when making mouse-specific predictions to determine the individual *α*_2_ for each mouse. Physiologically, this enables the development of a preliminary representation of the effectiveness of an individual dosing event (i.e., a specific administration interval of treatment) within the context of a larger treatment regimen. Resistance to treatment, as developed in our method, describes a progressive change in the treatment effect over multiple doses, which can manifest as either a relative reduction or an increase. Combination therapies involving stromal-targeting drugs display a higher level of resistivity to treatment than T1—NGC (Fig. 7). Since our model does not distinguish tumor volume between cancer cells and stroma, we hypothesize that the observed increase in resistivity may (in part) be attributed to stroma depletion caused by these drugs in the first treatment interval rather than a decrease in cancer cell count. Notably, our analysis indicates that the first delivery of treatment is between 0.72 and 4.5 times more effective than the second delivery of treatment. This indicates that there is a diverse level of resistance to treatment, dependent on the specific cocktail of therapies. Moreover, reverse resistance (i.e., when resistivity is less than 1.0) may be driven by several biological mechanisms such as stress priming^32^, delayed drug activation^33^, immune modulation^34^, tumor microenvironment remodeling^35^, epigenetic reprogramming^36^, or timing-dependent biological favorability^37^. Each of these mechanisms could contribute to the variability in treatment efficacy.

Our study contributes to the growing field of mathematical modeling in pancreatic cancer by developing a treatment-agnostic model that integrates multiple chemotherapy and stromal-targeting protocols and performs detailed sensitivity analyses to predict tumor response. This approach builds on the work of Hu et al.^21^, who employed a logistic growth assumption in their model of pancreatic cancer dynamics to explore the impact of immunotherapy on tumor progression. While Hu et al. focused on the interactions between tumor cells and the immune system, our model extends these ideas by evaluating the combined effects of various treatment protocols on tumor dynamics, including chemotherapy and stromal-targeting therapies. Additionally, our work complements that of Bratus et al.^20^, who explored the evolutionary dynamics of pancreatic cancer cells with a focus on genetic mutations and immune interactions. Whereas their model provides insights into tumor progression through genetic evolution, our model can predict treatment outcomes and generate hypotheses for guiding therapeutic strategies and exploring new combination therapies within the boundaries of the available data, but may be limited in its ability to extrapolate to scenarios involving significant changes in drug doses or schedules. Furthermore, in response to the gaps highlighted by Dogra et al.^17^, who pointed out the scarcity of mathematical models in pancreatic cancer, our study addresses the need for flexible models that can be adapted to different clinical scenarios. Lastly, while Chen et al.^38^ developed a PK/PD model focusing on the mechanistic behavior of gemcitabine, our treatment-agnostic approach allows for broader application across various therapeutic combinations, offering a framework for understanding how different treatment strategies can influence pancreatic cancer outcomes.

While our model shows accuracy and practical utility, there are several opportunities for improvement. First, our study modeled all chemotherapy drugs as a single entity, potentially overlooking specific interactions and efficacies of individual drugs. Future work should extend the model to account for the specific treatment protocols of each drug. For instance, in a previous study, we modeled six treatment protocols with two drugs, carefully estimating parameters of each using multiple measurements to capture individual and combined drug effects on breast cancer^39^. However, in this study, we chose a treatment-agnostic approach due to limited data points (three) and sought to assess whether the model could still capture overall tumor development trends without detailed protocol-specific information. Therapies for pancreatic cancer exhibit significant variation, particularly in their mechanisms of action and modes of application^40^. Thus, modifications to the mathematical framework—such as adding or removing parameters or adjusting the prior Bayesian distributions of existing parameters based on the specific mechanisms of each therapy—may improve model fit and enhance predictive power. Other frameworks for parameter estimation may be considered in future studies. Our current approach estimates the proliferation rate (*r*) on a mouse-by-mouse basis to capture inter-individual heterogeneity; this necessarily limits the sharing of information across treatment groups. Another area for improvement is the inclusion of pharmacokinetics in the model to account for drug delivery and distribution, which could provide deeper insights into treatment dynamics (as demonstrated in our previous work in breast cancer^41^). Even though post-processing of the parameter estimation results—by separating the parameter estimation of responders and non-responders and comparing the distributions of the parameters—revealed that non-responders tend to have higher proliferation rates and lower death rates due to treatment (Fig. 5), these differences are not accounted for in our current model. Incorporating a data assimilation approach, as demonstrated in ref. ^42^, could improve our model’s ability to dynamically update predictions based on new data. With the data assimilation approach, our model could be enhanced by differentiating between responders and non-responders earlier in the treatment process, thereby adapting the model parameters in real time to reflect these differences in proliferation and treatment response rates. This would allow for more personalized predictions and potentially more effective treatment strategies, ultimately improving the model’s predictive accuracy. Improvements in the model would, of course, require an increase in the amount and kind of data required to estimate parameters of the resulting model. Increasing the number of mice per treatment group would provide more responders and non-responders, aiding in better differentiation between these populations. It is important to note that in T4—calcipotriol and T5—NGC+calcipotriol + anti-PD-L1, every mouse is a responder; however, there are only four mice in T4—calcipotriol and eight mice in T5—NGC + calcipotriol + anti-PD-L1. Increasing the sample size in these groups would offer a more comprehensive understanding of the treatment effects. Furthermore, increasing the number of longitudinal data points beyond the three measurements currently available would improve the model’s predictive power and reliability.

Our mathematical model reproduces the overall dynamics of tumor progression and regression observed in a GEM model of pancreatic cancer treated with chemotherapies (cisplatin, paclitaxel, and gemcitabine) administered with or without stromal-targeting drugs (calcipotriol and losartan) and an immune checkpoint inhibitor (anti-PD-L1). Additionally, the model successfully predicts tumor volumes through several prediction schemes: mouse-specific predictions (CCC = 0.75 ± 0.05), leave-one-out predictions (CCC = 0.74 ± 0.14), and mouse-specific group-informed predictions (CCC = 0.85 ± 0.09). This work provides a rigorous mathematical framework for characterizing combination therapies for pancreatic cancer, particularly highlighting the interactions between chemotherapies, stromal-targeting drugs, and immunotherapy. To the best of our knowledge, this is the first mathematical model applied to pancreatic cancer that simultaneously (a) handles a six-drug combination spanning cytotoxic, stromal-targeting and immune-checkpoint agents, (b) supports principled model selection (via BIC) between constant-and decaying-effect hypotheses, and (c) demonstrates out-of-sample predictive accuracy (CCC ≈ 0.85) for responder classification. By accurately predicting responders and non-responders, the model can help tailor treatment strategies to individual subjects, potentially improving therapeutic outcomes. Additionally, the model’s ability to simulate various treatment regimens offers a valuable tool for exploring new combination therapies and optimizing existing ones.

## Methods

### Experimental design

All animal procedures were approved by the institutional animal care and use committee (IACUC) of the University of Pennsylvania. The experimental procedures for acquiring the tumor data are detailed more thoroughly in^43^.

The mouse model employed for these studies was a genetically engineered model (GEM) of pancreatic ductal carcinoma (${{Kras}}^{{\rm{LSL}}-{\rm{G12D}}}\,;\,{{Trp53}}^{{\rm{LSL}}-{\rm{R172H}}}\,;\,{Pdx1}-{\rm{Cre}}$, usually referred to as KPC mice^44–46^) bred at the Mouse Hospital of Abramson Cancer Center of the University of Pennsylvania. Notably, this KPC mouse model is immune competent, retaining an intact immune system that is essential for modeling tumor-immune interactions^47^. Both the male and female KPC mice were enrolled. Once their tumor reached 50–150 mm^3^ estimated by MRI, mice were randomized and assigned to one of the treatment groups described below.

**Fig. 8:**
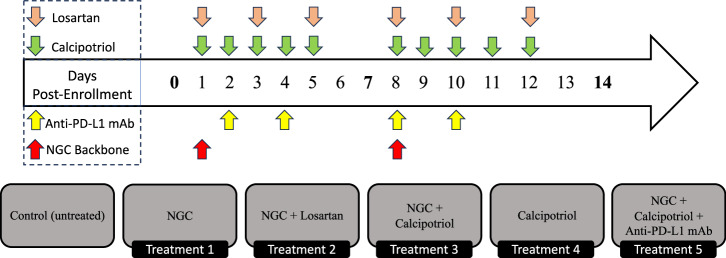
Depiction of the treatment regimes tested in the murine model of pancreatic cancer. Mice were treated for 14 days. Separate treatment components, namely NGC chemotherapy (red), anti-PD-L1 immunotherapy (yellow), and losartan (beige)/calcipotriol (green) stromal-targeting therapies, were included across five different treatment protocols (excluding control). Days with bolded time points represent tumor size measurements.

The treatment employed a chemotherapy backbone collectively referred to as NGC, consisting of Nab-paclitaxel (33 mg/kg), gemcitabine (266 mg/kg), and cisplatin (8 mg/kg for males and 4 mg/kg for females), stroma-directed drugs including calcipotriol (60 μg/kg) and losartan (30 mg/kg) and anti-PD-L1 mAb (200 μg/mouse). For the Nab-paclitaxel, murine albumin was used to develop murine albumin paclitaxel nanoparticles^48^. Six treatment groups were studied: control (untreated, *n* = 8), NGC (*n* = 18), NGC + losartan (*n* = 10), NGC + calcipotriol (*n* = 9), calcipotriol (*n* = 4), and NGC + calcipotriol + immunotherapy (anti-PD-L1 mAb; *n* = 8). All drugs were administered via intraperitoneal injection under isoflurane anesthesia, and all mice were euthanized by cervical dislocation under anesthesia on day 14 following MR imaging as described in ref. ^43^. The dosing schedule for each drug is described in Fig. 8.

To estimate the tumor volume, the tumor boundary was manually drawn on T2W images using the ImageJ software to generate tumor ROIs, and the areas from all ROIs was summed and the result was multiplied by the slice thickness. Tumor volume was assessed on day 0 prior to treatment, as well as on days 7 and 14 following treatment (Supplemental Fig. 4). Mice were sacrificed if a tumor measurement exceeded 1000 mm^3^.

### Mathematical models

To characterize tumor dynamics in response to targeted treatment, we propose the parameter estimation and prediction framework illustrated in Fig. 1. We begin by defining a set of ODEs to model the temporal dynamics of pancreatic tumor volume in response to various chemo- and targeted therapies. In particular, the model accounts for tumor proliferation, drug decay rate, tumor death rate, tumor carrying capacity, and initial tumor volume. Then, we compute a sensitivity analysis to identify the most important parameters (see visualization in Supplementary Figs. 5 and 6). Following this, we estimate the models’ parameters using in vivo experimental data using a Bayesian method to account for uncertainties in both the model and data. To compare the proposed models, we calculate the BIC and select the best model to undergo prediction analysis. Predictions are conducted in three separate scenarios: mouse-specific predictions, leave-one-out predictions, and mouse-specific group-informed predictions. To benchmark the effectiveness of these models as predictors, we evaluate their ability to successfully predict whether an individual mouse will become a treatment responder or not. We define responders as mice whose tumor volume at the end of the experimental regime is lower than their tumor volume at the start of treatment, and nonresponders are mice that do not fit this criterion. In this subsection, we will derive the models, while the other steps will be presented in subsequent subsections.

In our model, we assume the tumor volume at time *t*, *N*(*t*) increases logistically at a proliferation rate, *r*, up to a carrying capacity of *K* (see Table 1 for a listing of all model parameters):

2 $$\frac{dN}{dt}=rN\left(1-\frac{N}{K}\right)$$

To characterize the effect of treatment in our model, we introduce a compounding linear term, with an intensity of *α* to form our Linear Treatment Model:

3 $$\frac{dN}{dt}=rN\left(1-\frac{N}{K}\right)-N\alpha \mathop{\sum }\limits_{i=1}^{n}H(t-{\tau }_{i})$$

where *τ*_*i*_ is the time of the start of the *ith* treatment interval, *α* is the death rate due to treatment, *t* is the time post initial measurement (in days), and *H* is the Heaviside function. In this treatment-agnostic model, we do not account for each specific day the treatment was delivered. Instead, we focus on the compounding effects of the treatment between key time points; specifically, between the first and second measurements (*t*_1_ and *t*_2_, respectively) and between the second and third measurements (*t*_2_ and *t*_3_, respectively). Thus, we add the effect of the treatment at *τ*_1_ = *t*_1_ and *τ*_2_ = *t*_2_. Our Exponential Decay Treatment Model (Eq. (4)) extends the Linear Treatment Model (Eq. (3)), by incorporating a drug decay parameter, *β*, which allows the treatment term to exponentially decay mimicking the natural loss of drug concentration within the mice:

4 $$\frac{dN}{dt}=rN\left(1-\frac{N}{K}\right)-N\alpha \mathop{\sum }\limits_{i=1}^{n}{e}^{-\beta (t-{\tau }_{i})}H(t-{\tau }_{i})$$

### Bayesian method for parameter estimation

The in vivo longitudinal experimental data are utilized to estimate the parameters in our models (Eqs. (2) - (4)). To address the challenges posed by modest sample sizes and to rigorously account for parameter uncertainty, we have adopted a Bayesian method for parameter estimation. This probabilistic approach integrates prior information from control experiments and previous studies with our observed treatment data, thereby enhancing the robustness and interpretability of our parameter estimates (a similar approach has been taken in previous studies^39,49,50^). By explicitly quantifying uncertainties in both the model and the experimental measurements, we can generate reliable predictions of tumor dynamics under different therapeutic interventions. This method also strengthens our estimation process and supports the translational potential of our model by providing a rigorous basis for personalized treatment predictions. To implement this method, we define a log-likelihood function as

5 $$\ln \left(\pi ({\bf{D}}| {\boldsymbol{\theta }})\right)=-\frac{1}{2}\mathop{\sum }\limits_{i=1}^{{N}_{T}}\left[\ln \left(2\pi \right)\,+\,\ln \left({\sigma }^{2}\right)\,+\,\frac{{\left({D}_{i}-{Y}_{i}({\boldsymbol{\theta }})\right)}^{2}}{{\sigma }^{2}}\right],$$

where *i* is time point, *N*_*T*_ is the number of time points, ***D*** is the experimental data, ***θ*** is the vector of estimated model parameters, ***π***(***D***∣***θ***) is the likelihood that the data is observed for a set of parameters. To quantitatively differentiate between the performances of the models given by Eqs. (2), (3), and (4), we calculate the BIC^22^:

6 $${\rm{BIC}}=Z\ln (n)-2\ln (\widehat{L}),$$

where *Z* is the number of parameters estimated, *n* is the number of observed values, and $\hat{L}$ is the maximum value of the log likelihood. The model with the lowest BIC value represents the model that provides the most parsimonious description of the data, as it captures the highest likelihood of parameter value presence with a small penalty for the number of parameters, thereby prioritizing reductions in model complexity.

Our hierarchical framework allows for individualized variation for some parameters, making it conceptually similar to a mixed effects model. We note that certain parameters (e.g., proliferation rate, *r*, and initial tumor volume, *N*_0_) are estimated at the individual (mouse-specific) level, some are estimated at the treatment arm level (e.g., the decay of the drug, *β*), while others (e.g., carrying capacity, *K*) are estimated at the population level.

### Bayesian prior selection and prior predictive checks

In our Bayesian parameter estimation method, careful selection of prior distributions for the model parameters is essential to obtain reliable posterior inferences. To assess the suitability of these priors, we perform prior predictive checks, a simulation-based diagnostic that examines whether data simulated solely from the prior distributions are plausible in the context of our observed data. Let *θ* denote the vector of model parameters with prior distribution *p*(*θ*) and let *y* denote the observed data. The prior predictive distribution is given by

7 $$p(y)=\int\,p(y| \theta )\,p(\theta )\,d\theta ,$$

where *p*(*y*∣*θ*) is the likelihood defined by our tumor growth model. In practice, we approximate this integral via Monte Carlo simulation by drawing *N* samples ${\{{\theta }^{(i)}\}}_{i = 1}^{N}$ from the prior and, for each sample, simulating the corresponding data as

8 $${y}^{(i)}=f(t;{\theta }^{(i)}),$$

where *f*(*t*; *θ*) is the solution to the ordinary differential equation (either Eq. (3) or Eq. (4)). For each individual mouse, multiple simulated trajectories are generated using parameters sampled from the specified uniform priors (for example, $r \sim {\mathcal{U}}({r}_{\min },{r}_{\max })$, ${N}_{0} \sim {\mathcal{U}}(0,600)$, and $\alpha \sim {\mathcal{U}}(0,{a}_{\max })$). A coverage metric is calculated by determining the percentage of observed data points that lie within the central 95% predictive interval. This coverage diagnostic allows us to demonstrate that we are not over-restricting our prior domain. In our implementation, prior predictive checks are performed separately for the control group and for each of the five treatment arms. The five treatment arms are computed for both the Linear Treatment Model and the Exponential Decay Treatment Model. This comprehensive approach validates our choice of priors and ensures that our prior assumptions are capable of generating biologically realistic tumor dynamics prior to incorporating the data, thereby providing a solid foundation for subsequent Bayesian parameter estimation and model fitting.

### Sensitivity analysis

We utilize the Sobol method for performing a sensitivity analysis^51^, which is a variance-based measure that determines the relative effect of independent variables on the quantity of interest (e.g., tumor volume, *N*(*t*)). We apply this method via a sampling method described by Saltelli^52–54^, chosen for its efficiency in achieving convergence with a lower sample size. This application to our models is now described in detail (see Supplemental Fig. 7 for visual representation). Let **M**(***θ***) represent a model defined by *Z* parameters ***θ***, which reside in the parameter space ${\mathbf{\Theta }}\subset {{\mathbb{R}}}^{Z}$. We began by constructing matrices ***A*** and ***B*** by randomly sampling from a uniform distribution representing the uncertainty range of each parameter space.

Matrices ***A*** and ***B*** are of size *L* × *Z*, where *L* is the sample size length, and each row represents a unique set of parameters from the uncertainty space. Next, we develop *Z* matrices, ${{\boldsymbol{A}}}_{{\boldsymbol{B}}}^{(z)}$, *z* = 1, 2, 3, …, *Z* and all columns are duplicated from ***A*** except the *zth* column, which is copied from the *z*^*th*^ column of ***B***. The model is then solved for each row of matrices ***A*** and ${{\boldsymbol{A}}}_{{\boldsymbol{B}}}^{(z)}$ with the outputs stored in ***Y***_***A***_ and ${{\boldsymbol{Y}}}_{{\boldsymbol{AB}}}^{(z)}$, respectively, resulting in only *L* ⋅ (*Z* + 1) model evaluations. These outputs are used to evaluate ${S}_{{T}_{z}}$, the total sensitivity index, for each parameter, *z*. We approximate the total sensitivity index for each parameter using an estimator defined by Saltelli^54^:

9 $${{\rm{S}}}_{{{\rm{T}}}_{{\rm{z}}}}\approx \frac{1}{{\rm{2L}}}\mathop{\sum }\limits_{{\rm{j}}=1}^{{\rm{N}}}{\left({\left({{\rm{Y}}}_{{\rm{A}}}\right)}_{{\rm{j}}}-{\left({{\rm{Y}}}_{{\rm{AB}}}^{\left({\rm{z}}\right)}\right)}_{{\rm{j}}}\right)}^{2}$$

For dynamic processes (e.g., tumor growth), this form of sensitivity analysis allows us to evaluate the relative importance of individual model parameters at each time step. Thus, we can observe how the importance of each parameter changes over time to identify and eliminate unnecessary model parameters, thereby reducing model complexity. The sensitivity index is an approximation because it relies on a finite number of samples (*L*) to estimate the contribution of each parameter to the output variance. While this method provides a good estimate, the accuracy of the index improves with increasing *L*.

To facilitate a direct comparison of sensitivity indices between the linear and exponential treatment-response models, it is necessary to expose both systems to the same total “treatment pressure” over each dosing interval Δ*t*. In other words, we develop an adjustment to the sensitivity analysis method so that the parameter *α* represents similar physical quantities across the sensitivity analyses. In the linear model, the instantaneous death rate due to treatment is constant, *α*, so the cumulative effect over an interval Δ*t* is

$${E}_{{\rm{linear}}}\,=\,\alpha \,\Delta t.$$

In contrast, the exponential-decay model assumes that the instantaneous death rate decays at rate *β*, giving a time-varying rate *α* *e*^−*βt*^. The cumulative effect over Δ*t* is then

$${E}_{{\rm{expo}}}\,=\,\mathop{\int}\nolimits_{0}^{\Delta t}\alpha \,{e}^{-\beta \tau }\,d\tau \,=\,\frac{\alpha }{\beta }\,\left(1-{e}^{-\beta \Delta t}\right).$$

To compare these models on equal footing, we choose a scaling factor

$$s\,=\,\frac{\beta \,\Delta t}{1-{e}^{-\beta \Delta t}}$$

such that

$$s\,{E}_{{\rm{expo}}}\,=\,{E}_{{\rm{linear}}}\quad \Rightarrow \quad s\,\frac{\alpha }{\beta }\left(1-{e}^{-\beta \Delta t}\right)\,=\,\alpha \,\Delta t.$$

We therefore define an adjusted treatment parameter

$${\alpha }_{{\rm{adjusted}}}\,=\,s\,\alpha \,=\,\frac{\beta \,\Delta t}{1-{e}^{-\beta \Delta t}}\,\alpha ,$$

and throughout our sensitivity analysis we set Δ*t* = 7 days. In this way, both models receive the same total treatment effect each week, ensuring that differences in sensitivity indices arise solely from the distinct functional forms of treatment response rather than different physiological meanings for the treatment intensity parameter.

### Model predictions

We develop three separate prediction scenarios to test the translational potential of our model in a clinical application. In the first scenario, we estimate parameters for the first two time points of the available data for each mouse in each treatment group and then propagate the model solution forward to predict the tumor volume at the third and final time point for each mouse on an individual basis. The accuracy of the prediction is then compared to the experimental data via the concordance correlation coefficient (CCC). This mimics a clinical scenario in which we predict future tumor growth based on previous screenings without using heterogenous population data^55–57^. In the second scenario, we utilize a leave-one-out method and leverage population information to predict tumor volumes of individual mice in each treatment group. We began by constructing *M* matrices, ***C***_*m*_ with *m* = 1, 2, 3…*M*, where *M* is the number of mice in a treatment group. ***C***_*m*_ is defined by the observed tumor volume for the remaining *M* − 1 mice (i.e., excluding mouse *m*) in the treatment group at each time point. ***C***_*m*_’s rows are represented by different mice in the group, whereas the columns of this matrix represent time points of tumor volume measurement. All matrices ***C***_*m*_ are then used to run inference on the model using a Bayesian method. Then, to predict the tumor volume of a specific mouse, *m*, we randomly sample 1000 sets of parameters from the output space of the Bayesian parameter estimation (i.e., the posterior distribution) of ***C***_*m*_ to run our forward model. Thus, this method uses a set of 1000 parameters from the treatment group and the initial tumor volume to characterize a Bayesian distribution for future tumor volumes; namely, the tumor volumes at *t*_2_ and *t*_3_, where *t*_*i*_ represents the time of the *i*^*th*^ tumor volume measurement.

For scenario 3, we develop a hybrid of the mouse-specific and leave-one-out predictions. We began by splitting the drug effect into two separate linear summation terms, each representing the individual treatment doses administered on different days. This approach allows us to introduce a new correction factor for tumor-specific parameters and to account for the possibility that the tumor’s response to treatment may change after the first dose. For example, even if the doses are the same on both treatment days, the effect of the treatment might differ due to factors such as the tumor developing some level of resistance or other changes in its response over time. To account for this phenomenon, we modify the Linear Treatment Model as follows:

10 $$\frac{dN}{dt}=rN\left(1-\frac{N}{K}\right)-N{\alpha }_{1}H(t-{\tau }_{1})-N{\alpha }_{2}H(t-{\tau }_{2})$$

where *α*_1_ is the death rate due to treatment received between times *t*_1_ and *t*_2_, and *α*_2_ is the death rate due to the treatment received between times *t*_2_ and *t*_3_. As described in Fig. 8, we have five treatment combinations, and this same model is used for each treatment. Therefore, the parameters *α*_1_ and *α*_2_ can represent different treatments and combinations, with their values being estimated to match each specific treatment protocol. This allows us to develop relationships between treatment administration intervals. To account for the effect of serial doses, we make the simplifying assumption that *α*_2_ is proportional to *α*_1_, meaning we can describe *α*_2_ as a linear function of *α*_1_. The proportionality between *α*_2_ and *α*_1_, which we refer to as treatment resistance, is computed as the average ratio of *α*_1_ to *α*_2_ across the group of mice. To implement this concept of treatment resistance and predict the final tumor volume for mouse m, we begin by estimating parameters of the model given by Cumulative (Two-Dose) Linear Treatment Model, Eq. (10) to experimental data from every other mouse in the treatment group (i.e., *M* − 1), thereby determining the values of *α*_1_ and *α*_2_ for those mice. For mouse *m*, we then estimate only *α*_1_ and use the treatment resistance ratio from the group to estimate *α*_2_ (which has not been directly estimated for mouse *m*). This group-derived proportionality is applied to adjust *α*_2_ before continuing to forward propagate the model. Thus, for a single mouse, we combine our mouse-specific parameter estimation with the group-derived proportionality of compounding treatments to derive a prediction for the final tumor volume. Through this method, we effectively combine known group information regarding the effect of serial dosage while still maintaining mouse-specific modeling of tumors to create predictions.

### Numerical implementation

Eqs. (2) - (4) are implemented in Python 3.11.9, and the parameter estimation framework is illustrated in Fig. 1. The computation of the posterior density is performed through a parallel, adaptive, multilevel Markov Chain Monte Carlo sampling technique, available through the emcee 3.1.6 library^58^. We utilize 2*n* chains, where *n* is the parameter count, and run the simulation until we have 20,000 accepted samples per chain. The ODEs are solved using a fourth-order Runge-Kutta method. Detailed information on the code, including instructions on how to run it and the necessary dependencies, is available at https://github.com/krithikvishwanath.

### Statistics and reproducibility

Statistical analysis between Bayesian treatment groups is calculated using a two-tailed Mann-Whitney U test on tumor volumes. To do so, we assert that groups are independent of each other per experimental protocol. To evaluate the effects of growth between initial and final tumor volumes of a particular group (i.e., to demonstrate that the tumor is indeed growing in the control), we utilize a paired, two-tailed t-test. To assess statistical significance, a Bonferroni-adjusted *p*-value was used to maintain a 5% probability of a Type-I error. To quantify inter-individual variability in our calibrated parameters, we computed intra-class correlation coefficients (ICC) and coefficients of variation (CV) and report the results in Supplemental Table 1.

## Supplementary information

Supplementary Information

## Data Availability

The datasets generated and/or analyzed during the current study are available upon request to the corresponding author. Our code is shared publicly on GitHub upon publication of this work and can be found at https://github.com/krithikvishwanath/NGC_Therapy_Modeling.

## Code availability

Our code is shared publicly on GitHub upon publication of this work and can be found at https://github.com/krithikvishwanath/NGC_Therapy_Modeling.
