# Supplementary material for: Modeling tumor dynamics and predicting response to therapies in a murine pancreatic cancer model
Source: NPJ Syst Biol Appl. 2025 Nov 4;11:123. doi: 10.1038/s41540-025-00593-z (PMC12586507; doi:10.1038/s41540-025-00593-z)
Supplement: Supplementary file 1 — Supplementary Information [file 41540_2025_593_MOESM1_ESM.pdf]

Supplementary Material

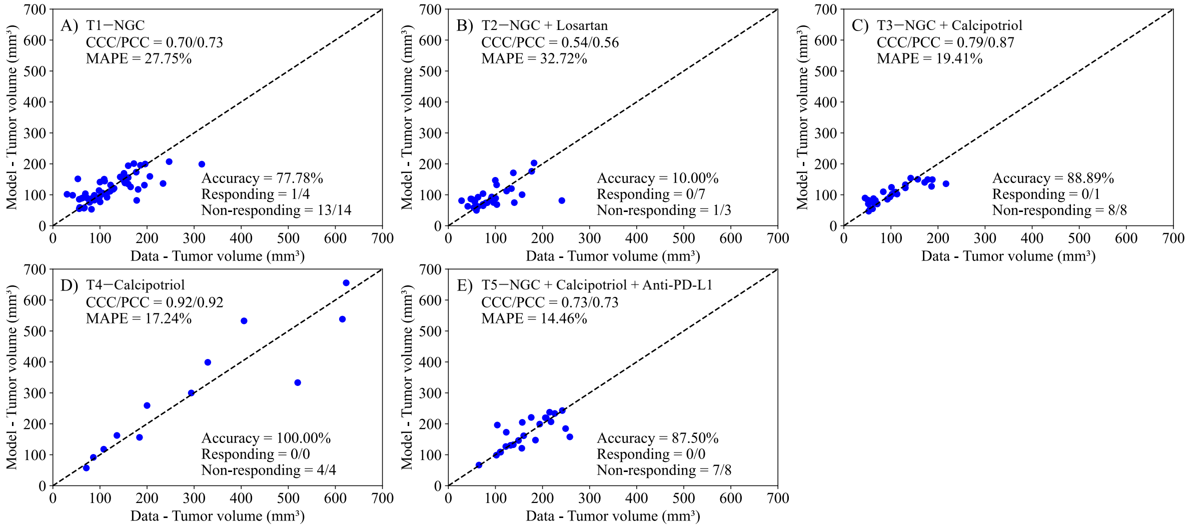

Supplemental Figure 1. Comparison of tumor volume from experimental data and tumor volume from leave-one-out predictions for each treatment scenario. The dashed black lines are the line of unity. The average accuracy was 72.83 ± 16.10% across all treatment scenarios. Notably, leave-one-predictions exhibited greater than 75% accuracy of correctly differentiating between responders and non-responders, and a CCC and PCC greater than 0.7 for all scenarios other than Treatment 2. The MAPE is below 33% for all scenarios, performing with the lowest percent error for Treatment 5 (MAPE = 14.75%) and the greatest for Treatment 2 (MAPE = 32.22%).

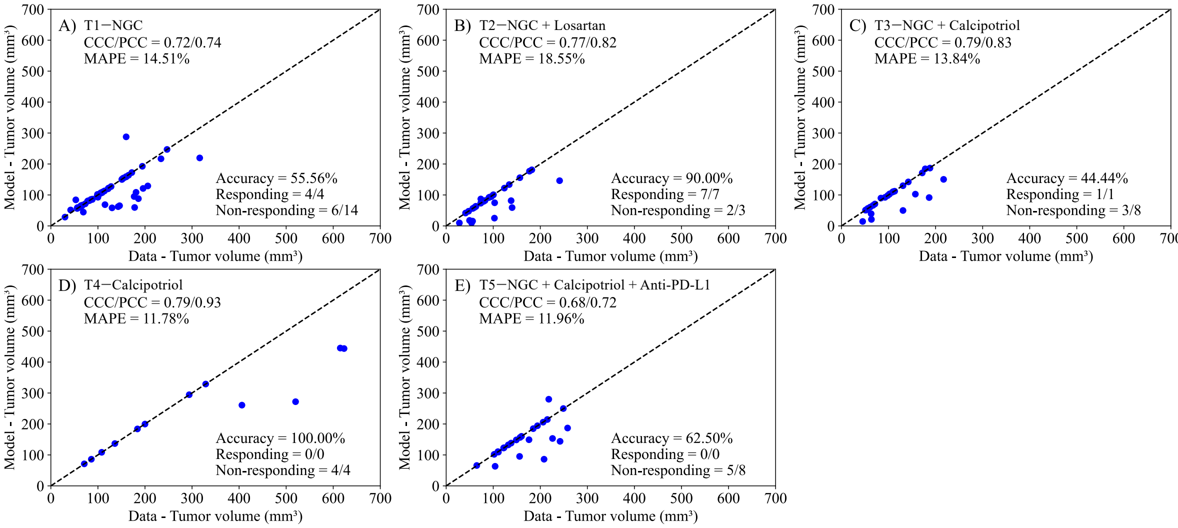

Supplemental Figure 2. Comparison of tumor volume from experimental data and tumor volume from mouse-specific predictions for each treatment scenario. The dashed black lines are the line of unity. The average accuracy was 70.50 ± 10.53% across all treatment scenarios. Mouse-specific predictions exhibited a concordance correlation coefficient (CCC) and Pearson correlation coefficient (PCC) greater than 0.68 for all scenarios. The mean absolute percent error (MAPE) is below 18.54% for all scenarios, performing with the lowest mean percent error for Treatment 5 (MAPE = 11.96%) and the greatest mean percent error for Treatment 2 (MAPE = 18.55%).

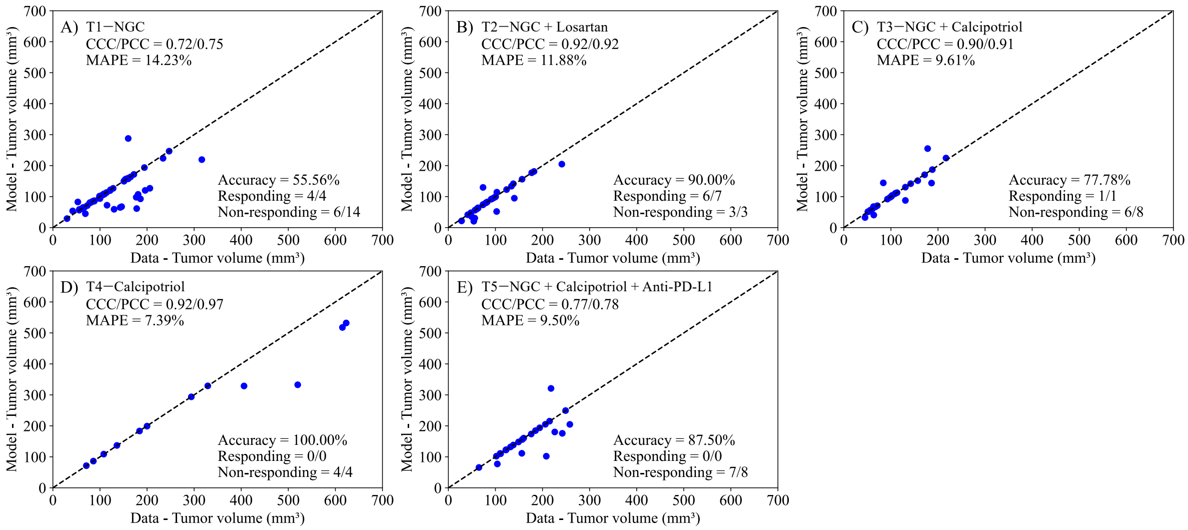

Supplemental Figure 3. Comparison of tumor volume from experimental data and tumor volume from group-informed, mouse-specific predictions for each treatment scenario. Calibrations utilize the population resistivity dynamics to predict tumor volumes for specific mice within the population. The dashed black lines are the line of unity. The average accuracy was 82.17 $\pm$ 7.53% across all treatment scenarios. Group-informed, mouse-specific predictions exhibited a CCC and PCC greater than 0.72 in all scenarios. The MAPE is below 14.32% for all scenarios, performing with the lowest mean percent error for Treatment 4 (MAPE = 7.29%) and the greatest mean percent error for Treatment 1 (MAPE = 14.32%).

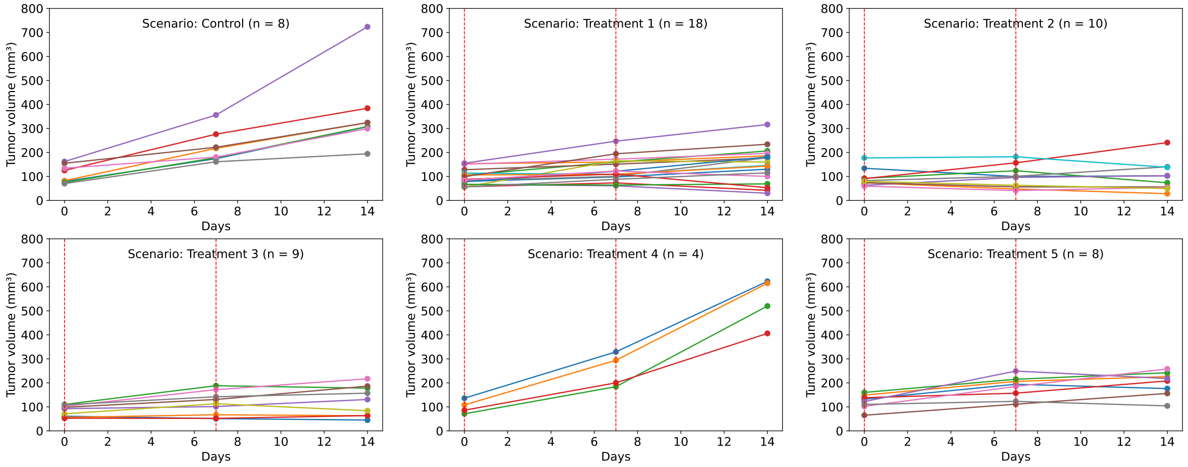

Supplemental Figure 4. Full data depiction of the tumor volume, and their tumor dynamics across the experimental regime (day 0 to day 14). Tumors in groups 1 and 4 demonstrate a visibly steeper increase tumor volume.

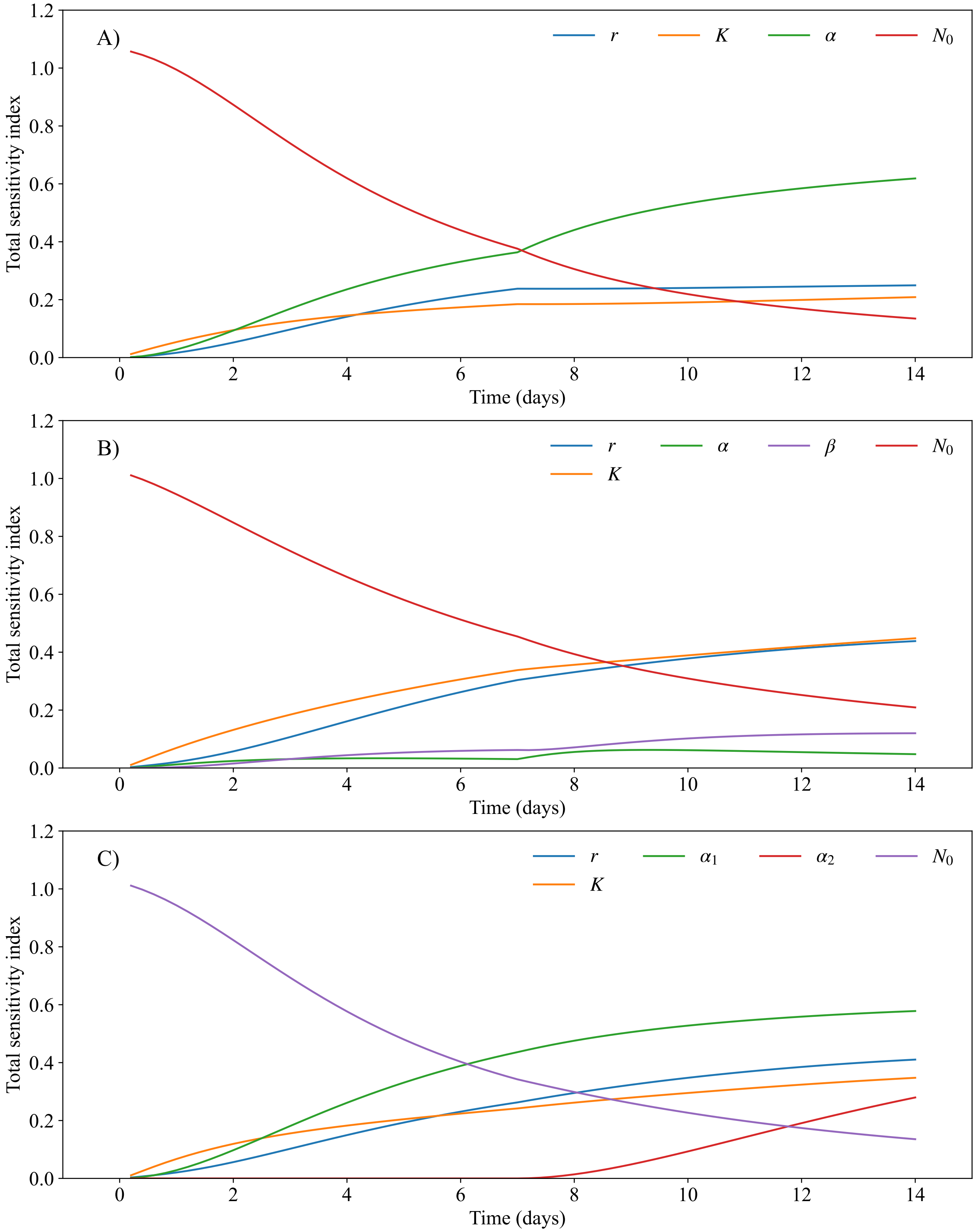

Supplemental Figure 5. Sensitivity analysis of the Linear Treatment Model (Eq. (2); panel A), the Exponential Decay Treatment Model (Eq. (3); panel B), Cumulative (Two-Dose) Linear Treatment Model (Eq. (9); panel C). The total sensitivity index (Eq. (4)) is computed for each model, demonstrating how the parameters affect tumor volume throughout the experimental time course. In both panels, the initial tumor volume plays a large role before the second dose of treatment (day 7), but

its effect reduces as the experiment continues. In panel A, the death rate due to treatment (α, green line) has the highest total sensitivity index by far (approximately $3\times$ higher than the second most influential parameter), followed by the proliferation rate. However, for panel B, the carrying capacity (K, orange line) and proliferation rate (r, blue line) exhibit nearly identical total sensitivity indices by day 14, with similar temporal dynamics throughout the experiment. Both parameters emerge as the most influential factors after day 9. These parameters are followed in total sensitivity index by the initial tumor volume (N_0, red line). This discrepancy between panel A and panel B's high-order parameters is likely due to the lack of decay present for the treatment in the panel A model, resulting in a larger value of the death rate due to treatment parameter, α.

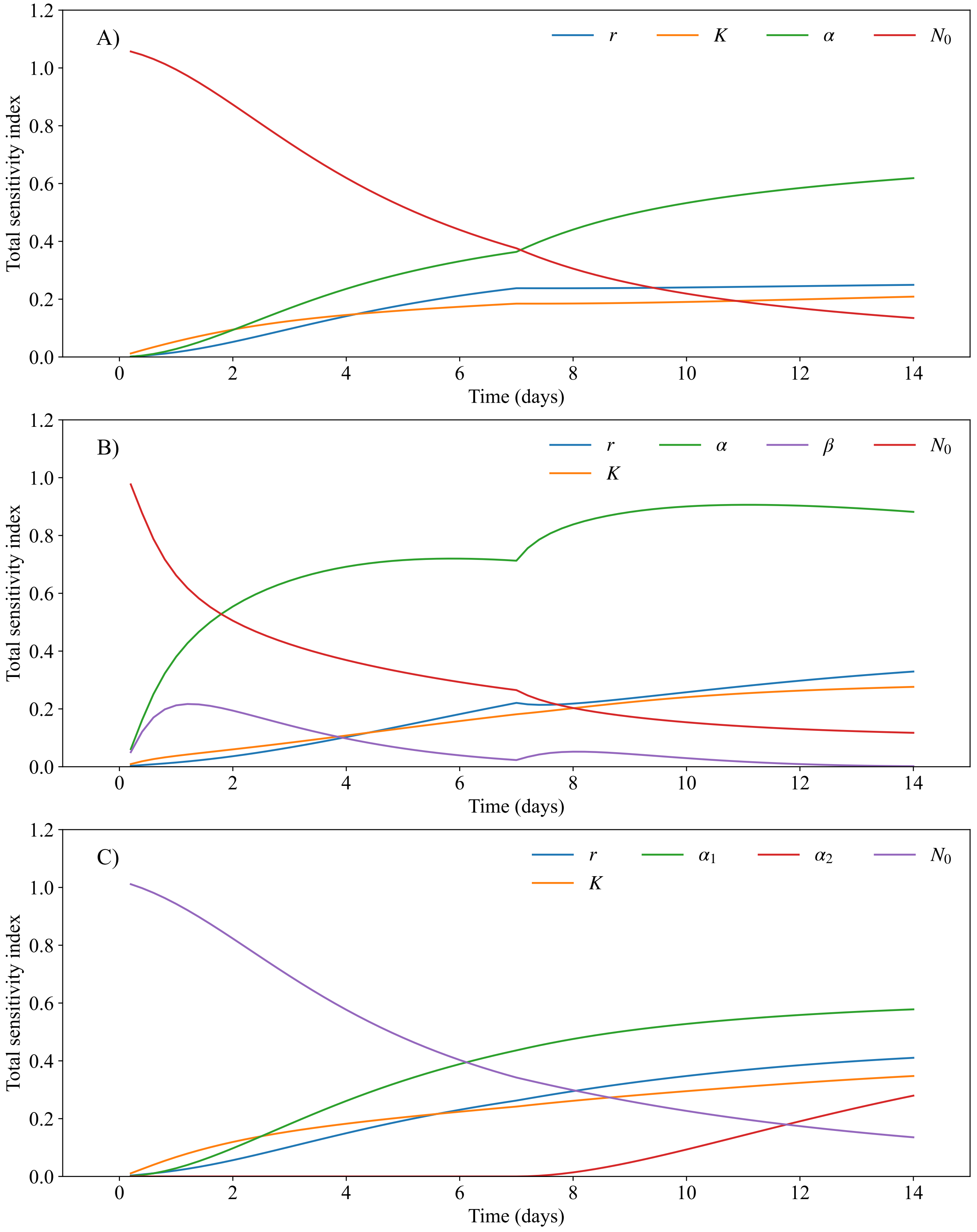

Supplemental Figure 6. Sensitivity analysis of the Linear Treatment Model (Eq. (2); panel A), the Exponential Decay Treatment Model with an adjusted alpha parameter (Eq. (3); panel B), Cumulative (Two-Dose) Linear Treatment Model (Eq. (9); panel C). The total sensitivity index (Eq. (4)) is computed for each model, demonstrating how the parameters affect tumor volume throughout the experimental time course. In both panels, the initial tumor volume plays a large role before the second

dose of treatment (day 7), but its effect reduces as the experiment continues. In panel A, the death rate due to treatment (α, green line) has the highest total sensitivity index by far (approximately $3\times$ higher than the second most influential parameter), followed by the proliferation rate. However, for panel B, the carrying capacity (K, orange line) and proliferation rate (r, blue line) exhibit nearly identical total sensitivity indices by day 14, with similar temporal dynamics throughout the experiment. Both parameters emerge as the most influential factors after day 9. These parameters are followed in total sensitivity index by the initial tumor volume (N_0, red line). This discrepancy between panel A and panel B's high-order parameters is likely due to the lack of decay present for the treatment in the panel A model, resulting in a larger value of the death rate due to treatment parameter, α.

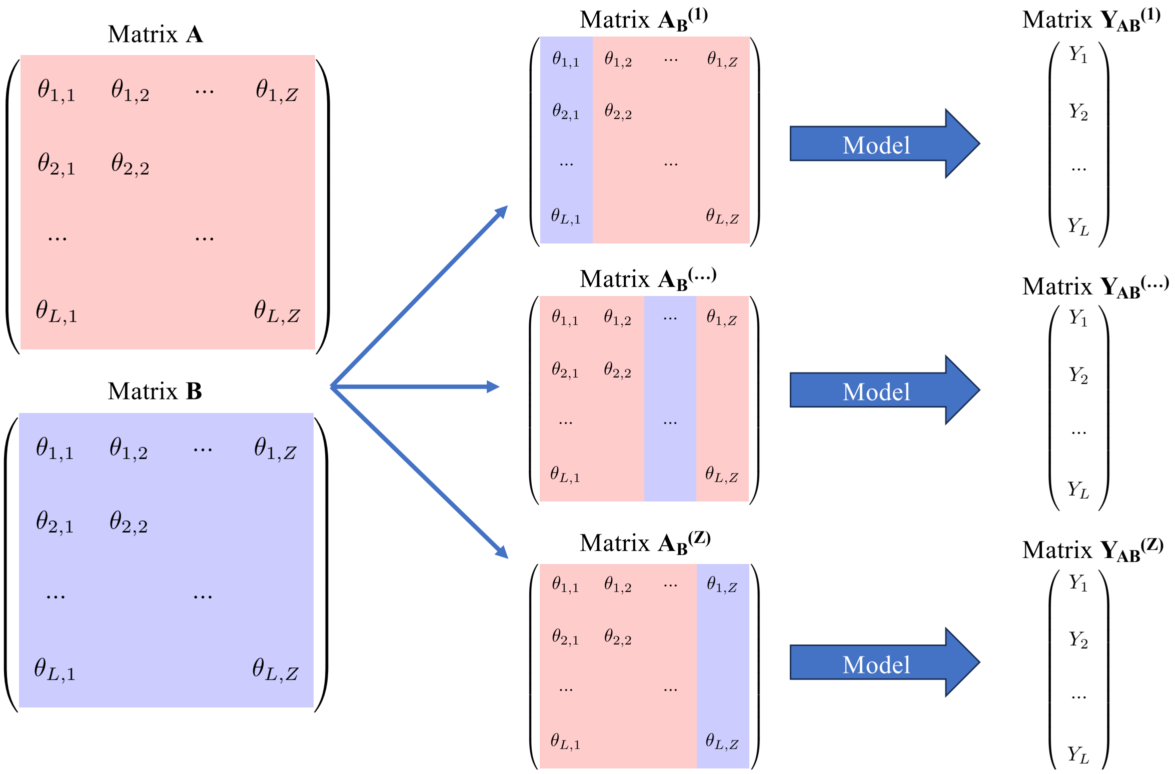

Supplemental Figure 7. Visual representation of total sensitivity index estimation using matrix notion. Theta, $\boldsymbol{\theta}$, is defined as a vector of parameters for a particular model. We began by developing matrices $\boldsymbol{A}$ and $\boldsymbol{B}$, where arbitrary column z is a vector of L values randomly generated from the sample space of the zth parameter. $\boldsymbol{A}$ and $\boldsymbol{B}$ are then used as components of a new hybrid matrix, with one column from $\boldsymbol{B}$ and the rest from $\boldsymbol{A}$. Then, the model is propagated for each hybrid matrix and for matrix $\boldsymbol{A}$. Variations between the output of matrix $\boldsymbol{A}$ compared to each hybrid matrix allow insight of parameter influence on model output, within the sampling space.

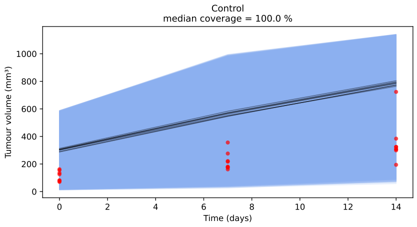

Supplemental Figure 8. Results from prior predictive checks on the Logistic Growth Model. Prior predictive checks on the control mice using the Logistic Growth Model achieve 100% coverage, defined as the percentage of observed data points that lie within the central 95% predictive interval.

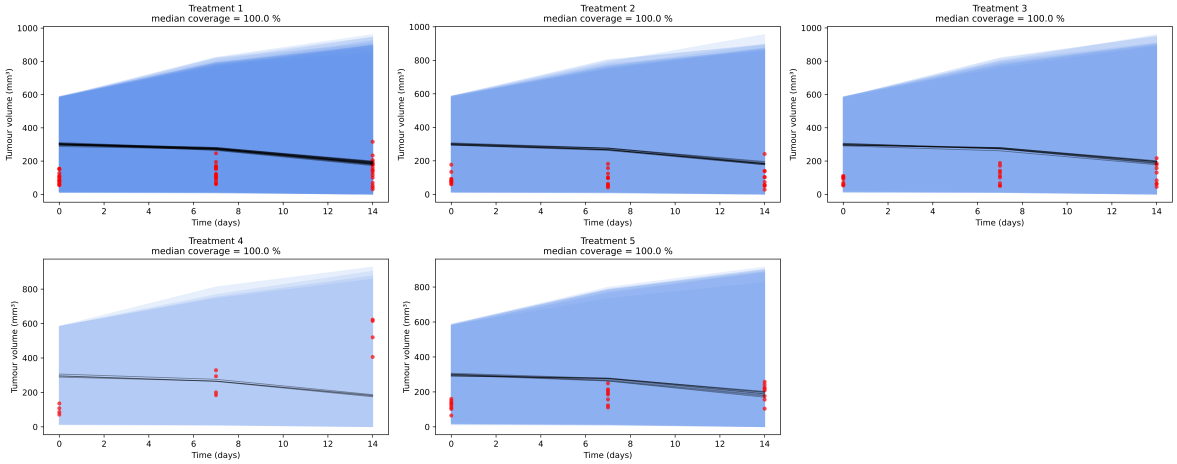

Supplemental Figure 9. Results from prior predictive checks on the Linear Treatment Model. Prior predictive checks on all treatment scenario mice using the Linear Treatment Model achieve 100% coverage, defined as the percentage of observed data points that lie within the central 95% predictive interval.

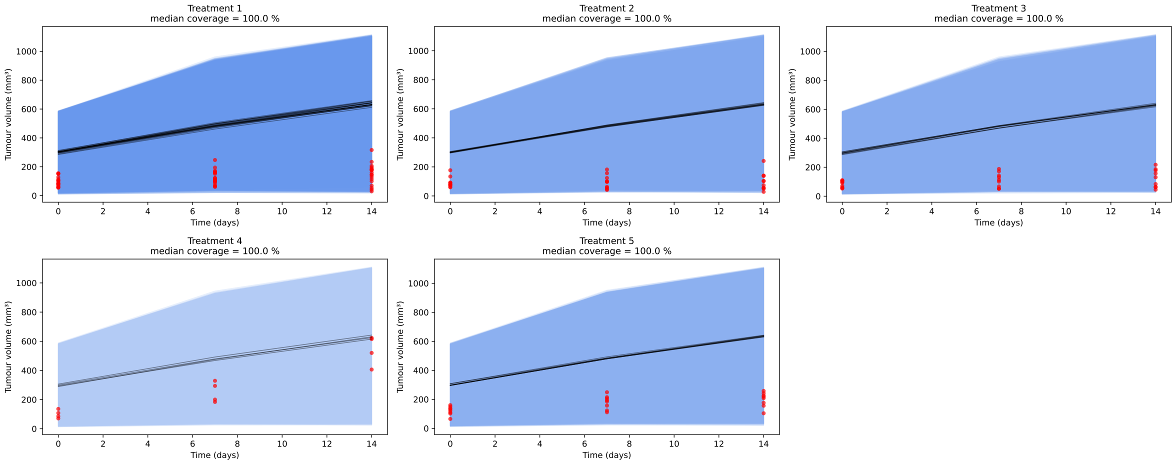

Supplemental Figure 10. Results from prior predictive checks on the Exponential Decay Treatment Model. Prior predictive checks on all treatment scenario mice using the Exponential Decay Treatment Model achieve 100% coverage, defined as the percentage of observed data points that fall within the simulated outcomes.

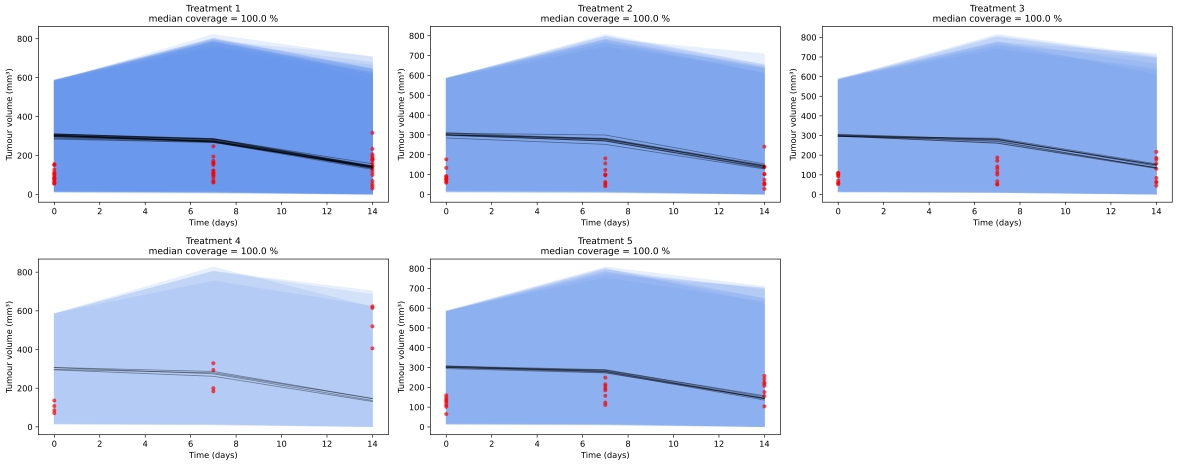

Supplemental Figure 11. Results from prior predictive checks on the Cumulative Linear Treatment Model. Prior predictive checks on all treatment scenario mice using the Cumulative Linear Treatment Model achieve 100% coverage,defined as the percentage of observed data points that lie within the central 95% predictive interval.

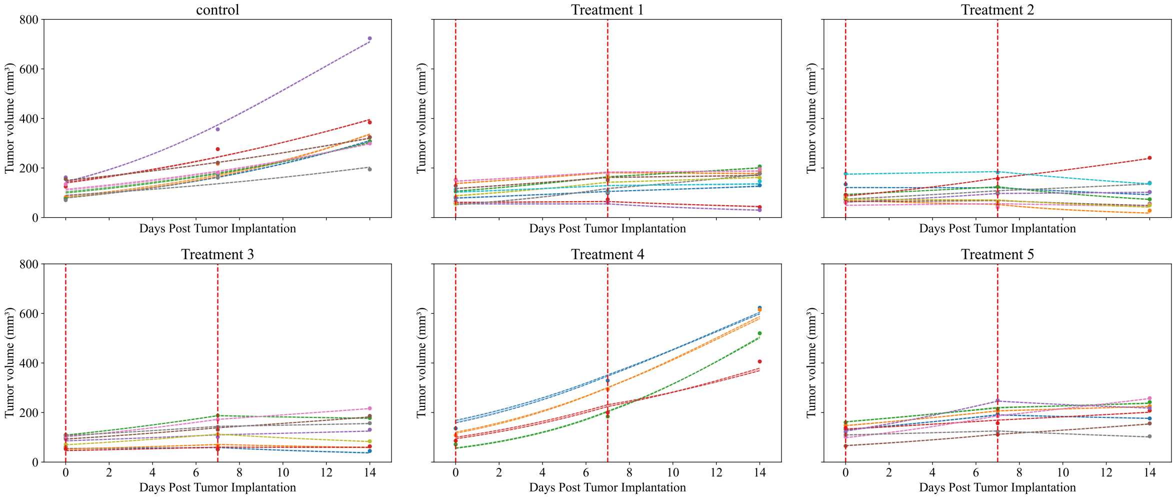

Supplemental Figure 12. Model fittings plotted on top of time series data. Dashed red vertical lines indicate treatment administration.

Table 1: Intra-class correlation coefficients (ICCs) and coefficient of variation (CV) across the entire set of mice in any treatment group.

**Parameter**	**ICC**	**CV**
Initial tumor buden (IC)	0.91	50%
Treatment effect (α)	0.97	3.3%
Proliferation rate (r)	0.60	45%
